# Ultrahigh Frequency Ultrasonic Transducers (150MHz) Based on Silicon Lenses

**DOI:** 10.3390/mi14010213

**Published:** 2023-01-14

**Authors:** Jun Chen, Chunlong Fei, Jianxin Zhao, Yi Quan, Yecheng Wang, Zhishui Jiang, Li Wen

**Affiliations:** 1 School of Microelectronics, Xidian University, Xi’an 710071, China; 2 Guangdong JC Technological Innovation Electronics Co., Ltd., Zhaoqing 526000, China

**Keywords:** silicon lens, acoustic lens, ultrahigh frequency ultrasonic transducer, ultra-precision machining, acoustic microscope

## Abstract

Acoustic microscopes and acoustic tweezers have great value in the application of microparticle manipulation, biomedical research and non-destructive testing. Ultrahigh frequency (UHF) ultrasonic transducers act as the key component in acoustic microscopes, and acoustic tweezers and acoustic lenses are essential parts of UHF ultrasonic transducers. Therefore, the preparation of acoustic lenses is crucial. Silicon is a suitable material for preparing acoustic lenses because of its high acoustic velocity, low acoustic attenuation and excellent machinability. In previous research, silicon lenses were mainly prepared by etching. However, etching has some drawbacks. The etching of large sizes is complex, time-consuming and expensive. Furthermore, vertical etching is preferred to spherical etching. Thus, a new method of ultra-precision machining was introduced to prepare silicon lenses. In this paper, silicon lenses with an aperture of 892 μm and a depth of 252 μm were prepared. Then, UHF ultrasonic transducers with a center frequency of 157 MHz and a −6-dB bandwidth of 52% were successfully prepared based on silicon lenses. The focal distance of the transducers was 736 μm and the F-number was about 0.82. The transducers had a lateral resolution of 11 μm and could distinguish the 13 μm slots on silicon wafers clearly.

## 1. Introduction

Ultrasonic energy can penetrate the interior of objects and measure their elastic properties through echo without damaging them. Therefore, ultrasound can be used to image the interior of objects. Ultrasonic imaging has the advantages of being real-time, inexpensive and harmless to organisms. Acoustic microscopes [1,2,3,4,5,6,7,8,9] are the systems used for high resolution ultrasonic imaging. Acoustic microscopes have great value in applications such as biomedical imaging [3,4,5,10,11] and non-destructive testing [7,8,9,12,13]. The resolution of systems is determined by the working frequency of transducers. Thus, ultrahigh frequency (UHF) ultrasonic transducers [10,14] are the key components in acoustic microscopes.

When particles are suspended in a field of acoustic waves, the acoustic radiation force [15] that arises from the scattering of the acoustic waves is exerted on particles. The contactless manipulation of particles can be realized by acoustic waves. The systems that manipulate the position and movement of very small objects with acoustic waves are called acoustic tweezers. Acoustic tweezers [16,17] have numerous applications in biophysical and biomedical research fields [16,17,18]. The target objects are usually smaller than the wavelength of acoustic waves used. The use of UHF ultrasound transducers allows for extremely high manipulation accuracy, so UHF ultrasonic transducers are crucial to acoustic tweezers.

Ultrasonic transducers with dispersed acoustic beams are difficult to be apply in imaging or manipulation. The acoustic beams need be focused to obtain high resolution and energy density. Acoustic lenses [19,20,21] can be applied for focusing acoustic beams and are an essential part of ultrasound transducers. At low frequency, there are few limitations for preparing acoustic lenses, but for UHF frequency, the attenuation of acoustic energy is proportional to the square of the frequency. Thus, the size of acoustic lens is considerably decreased, and the difficulty of its preparation is much increased. Therefore, suitable materials and accurate processing methods are important for the preparation of UHF lenses.

Silicon is a suitable material for preparing UHF acoustic lenses because of its high acoustic velocity, low acoustic attenuation and excellent machinability. In previous research, silicon lenses were prepared by etching. The etching [14,18,22] of silicon is a common method in the field of semiconductors, although it is expensive and complex. Etching involves a series of processes including coating, exposure, development, etching, and striping. Thus, low-cost and efficient methods are needed to simplify the preparation of UHF acoustic lenses. In the following section, the preparation of UHF acoustic lenses by a new method is described. Then, the design and simulation of UHF acoustic lenses are presented. Finally, the preparation of UHF ultrasonic transducers is described, and characterization and imaging experiments are discussed.

## 2. Materials and Methods

### 2.1. Design, Simulation and Fabrication of Silicon Lens

**Figure 1:**
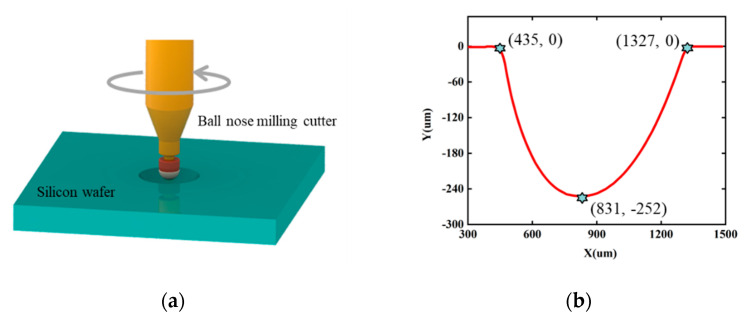

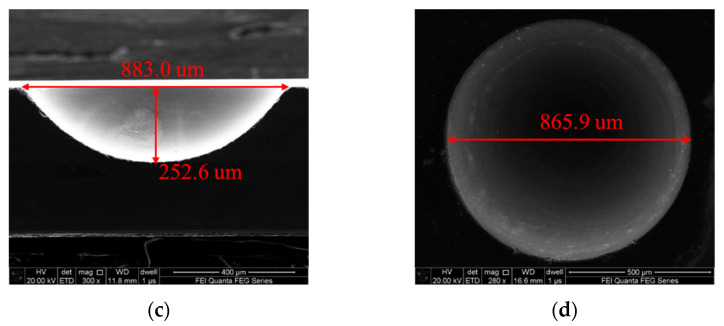
(**a**) Spherical holes machining. (**b**) Contour of the silicon lens measured by stylus profilometer. (**c**) A cross-sectional view of the silicon lens imaged by SEM. (**d**) A top view of the silicon lens imaged by SEM.

Ultraprecision machining was applied for preparing silicon lenses. The process utilized an ultraprecision high-speed Micro Machining Center (QJM-VL1S, Quick Jet, Kunshan, China) and ball nose milling cutter (EPDBEH-TH3 series, Hitachi, Tokyo, Japan) with a diameter of 100 um (Figure 1a). 3D models of the lens were required for automatic machining. Spherical holes were machined on the surface of the silicon. Diamond polishing paste of 1 micron was then used to obtain a superfine mirror finish. The surface undulation of the lens was less than 1 μm. The contour measured by stylus profilometer (DektakXTL, Bruker, Massachusetts, MA, USA) demonstrated the result of polishing (Figure 1b). The morphology of silicon lenses can be observed in a scanning electron microscope (SEM). Figure 1c and d show the cross-sectional and top view of a silicon lens captured by SEM. The size of the silicon lens was measured using the ruler tools in the SEM software. The silicon lens showed a complete spherical morphology. The parameters of the silicon wafer used in this process are shown in Table 1.

**Figure 2:**
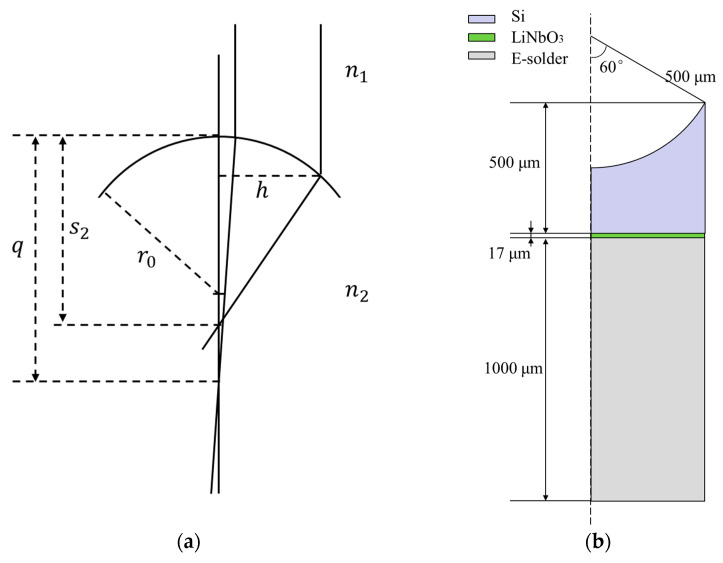
(**a**) Schematic diagram of lens design;(**b**) 2D axisymmetric model of transducers based on a silicon lens.

Acoustic lenses with a large focal distance have a wide working range, while acoustic energy attenuation also increases with distance. Thus, the design of focal distance should not be too large when the depth of applications is low. The focal distance (q) of silicon lenses can be calculated by Formula (1) [19,23]. When the incident acoustic waves are a distance (h) from the center axis, q is changed to $s_{2}$ (Figure 2a), and this aberration decreases the resolution. According to Formula (2) [23], the aberration of the focal point is proportional to the square of aperture. Although a large aperture can allow more energy to penetrate, the size of the aperture should be considered seriously. The F-number [19] is defined as the focal distance divided by the aperture. An appropriate value of the F-number can balance the acoustic energy and resolution. An F-number of 0.8 [19] was chosen for the design of silicon lenses. To avoid edge effects, the aperture of lenses should be less than the size of the piezoelectric materials. The size of the piezoelectric materials in UHF ultrasonic transducers was 1000 × 1000 μm. Thus, the aperture of silicon lenses was designed to 900 μm and the focal distance was 720 μm. In addition, $s_{2}$ is inversely proportional to the square of n. A small value of n leads to small aberration. The commonly used coupling medium is water with an acoustic velocity of 1540 m/s. Silicon has an acoustic velocity of 8430 m/s, which is much larger than that of water. The value of *n* was about 0.18, as calculated by Formula (3) [23]. Thus, silicon is a suitable material for preparing acoustic lenses. The lens properties are calculated as follows:

(1) $$q=\frac{r_{0}}{1-n}\;$$

(2) $$\frac{1}{s_{2}}=\frac{1}{q}+\frac{n^{2}h^{2}}{2q{r_{0}}^{2}}\;$$

(3) $$n=\frac{n_{1}}{n_{2}}=\frac{v_{2}}{v_{1}}\;$$

where $r_{0}$ is the radius of the curvature, $n_{1}$ is the refractive index of the lens, $n_{2}$ is the refractive index of the medium, $v_{1}$ is the acoustic velocity in lens, and $v_{2}$ is the acoustic velocity in the medium.

**Figure 3:**
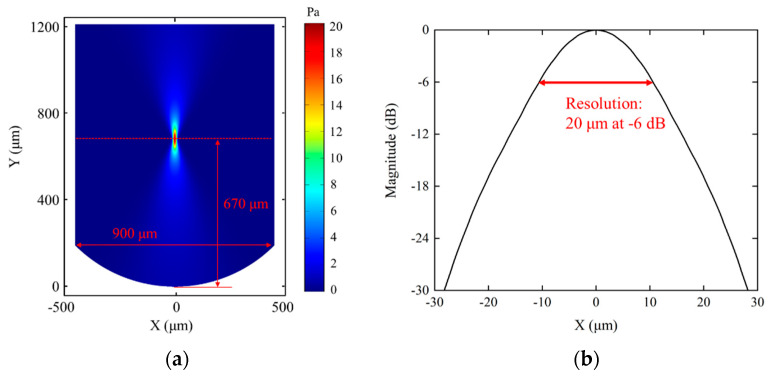
(**a**) The acoustic pressure field simulated by COMSOL. (**b**) The lateral resolution simulated by COMSOL.

To verify the effectiveness of the silicon lens, a commercial finite element method software (COMSOL Multiphysics 6.0) was used to establish a 2D finite element model based on Figure 2b. The silicon lens was symmetrically modeled with a depth of 250 μm. Silicon was selected to fabricate the lens, and a LiNbO_3_ crystal was selected as the piezoelectric material because of its excellent acoustic properties. The acoustic parameters used for the simulation are listed in Table 2. A highly-attenuated conductive epoxy (E-solder 3022) was selected as the backing to reduce the rings from the back of the piezo-element. The physical fields (pressure acoustics, solid mechanics, and electrostatic mechanics) were selected to accurately simulate the propagation of acoustic waves. Silicon and water were attributed to the pressure acoustics field, and LiNbO_3_ was assigned to solid mechanics and electrostatic mechanics. To simulate the infinite water domain and avoid the influence of non-experimental factors, a perfect matching layer was selected on the outermost side of the model. Finally, a sufficiently small mesh (less than λ/5, λ means the wavelength at the center frequency) was divided to obtain more accurate simulation results, and the frequency sweep range of 100–200 MHz with step of 5 MHz was applied to simulate the acoustic field under different excitation frequencies. Figure 3a shows the simulated acoustic pressure intensity distribution in water. Furthermore, the lateral resolution could be calculated by the transversal line past the center of the focal point. As shown in Figure 3b, the lateral resolution was 20 μm at −6-dB magnitude.

### 2.2. Fabrication of UHF Ultrasound Transducer

**Figure 4:**
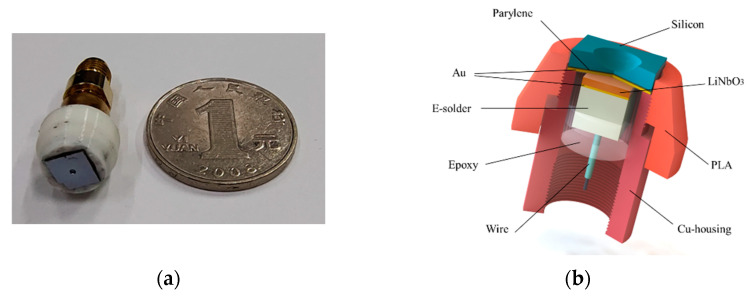

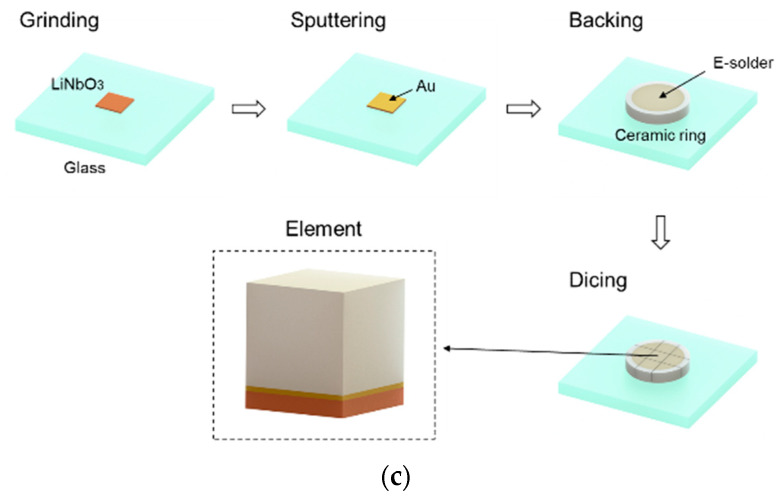
(**a**) The fabricated devices. (**b**) Schematic diagram of UHF ultrasound transducers. (**c**) The preparation processes of the elements.

As shown in Figure 4c, a piece of LiNbO_3_ single crystal was stuck on the glass substrate with paraffin and was ground to 17 μm. An Au-layer with a thickness of 200 nm was sputtered (Desk V, Denton Vacuum, Moorestown, NJ, USA) on the surface of the LiNbO_3_. A ceramic ring was used for filling the E-solder 3022 conductive adhesive, and E-solder 3022 (Von Roll Isola Inc., Schenectady, NY, USA) was ground to 1 mm as a backing layer. The elements were diced into 1 × 1 mm by a dicing saw (DAD323, Disco, Japan). Then, UHF ultrasound transducers were packaged according to Figure 4b. The bottom electrode was connected by the backing layer and wire. The element was then placed in the Cu-housing. The Cu-housing was filled with epoxy to fix the element. An Au-layer with a thickness of 200 nm was sputtered on the surface of the transducers as the top electrode and connected the Cu-housing. A parylene layer with a thickness of 3 μm (PDS 2010, Specialty Coating Systems, Indianapolis, IN, USA) acted as a protective film. The silicon lens was fixed in a PLA 3D printing mold. The mold and Cu-housing were assembled by threads, and a couplant was used for removing the air between the silicon lens and the element. The fabricated devices are shown in Figure 4a.

## 3. Results

### 3.1. Characteristics of Transducers

**Figure 5:**
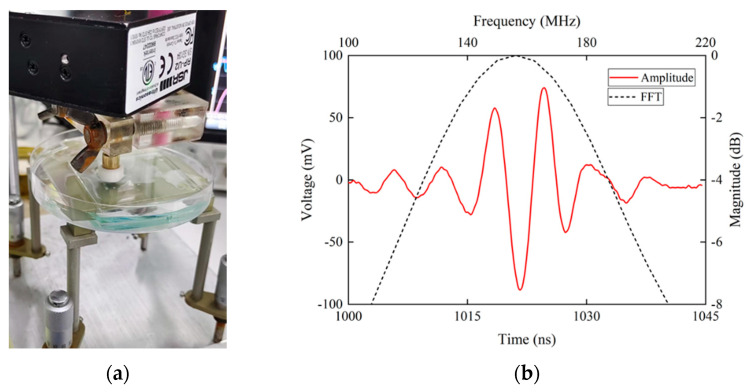
(**a**) Pulse-echo testing. (**b**) The waveform and frequency spectrum of the echo.

The pulse-echo experiment is commonly used for characterizing ultrasonic transducers. Here, the UHF ultrasonic transducers were characterized by pulse-echo testing. A piece of glass acted as reflector (Figure 5a). JSR (DPR500, Imaginant, Pittsford, NY, USA) was used to transmit a pulse and receive an echo. The results are shown in Figure 5b, and the relevant parameters are listed in Table 3. The center frequency of the transducers determines the physical limit of resolution. The −6-dB bandwidth affects the waveform of echoes. Pulse duration was used for describing the length of the echo and is defined by the duration of the half amplitude of the echoes. The performance of LiNbO_3_ transducers without a lens has been shown in previous research [10,24,25,26].

### 3.2. B-Scan Imaging

**Figure 6:**
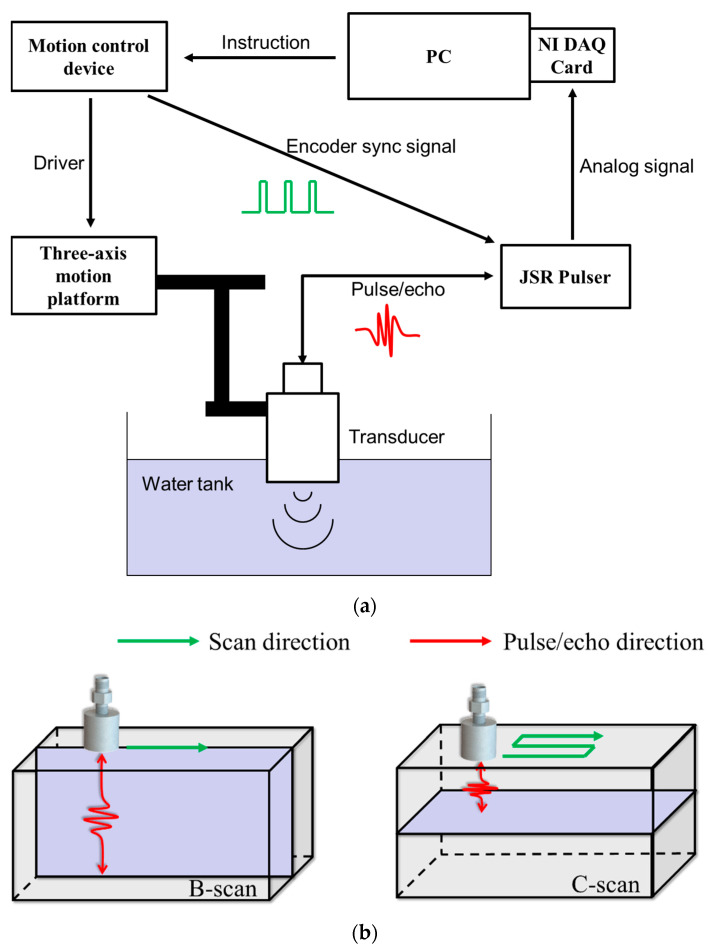
(**a**) Schematic diagram of scanning acoustic microscope (SAM). (**b**) Schematic diagram of B-scan and C-scan.

Scanning acoustic microscope (SAM) usually refers to ultrasound imaging devices consisting of single-element ultrasound transducers, three-axis motion platforms, and a data acquisition (DAQ) card. Figure 6a shows a schematic diagram of an SAM. Figure 6b shows the general working modes of an SAM.

**Figure 7:**
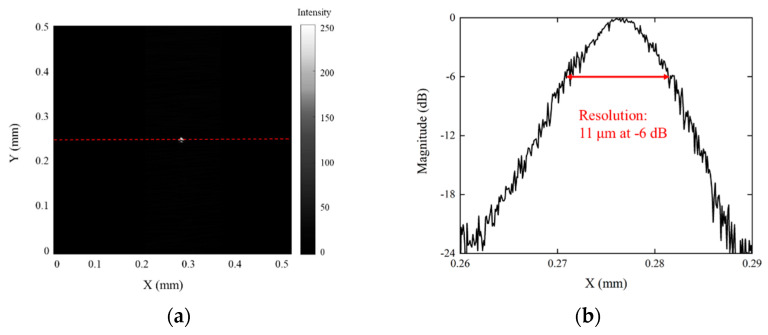
(**a**) An ultrasonic image of the 10μm tungsten wire. (**b**) The lateral resolution of the UHF ultrasonic transducer.

Tungsten wires with a diameter of 10 μm were imaged by SAM. The tungsten wires could be distinguished in the B-scan image (Figure 7a). The lateral resolution could be calculated by the transversal line past the center of the tungsten wires. As shown in Figure 7b, the resolution was 11 μm.

### 3.3. C-Scan Imaging

**Figure 8:**
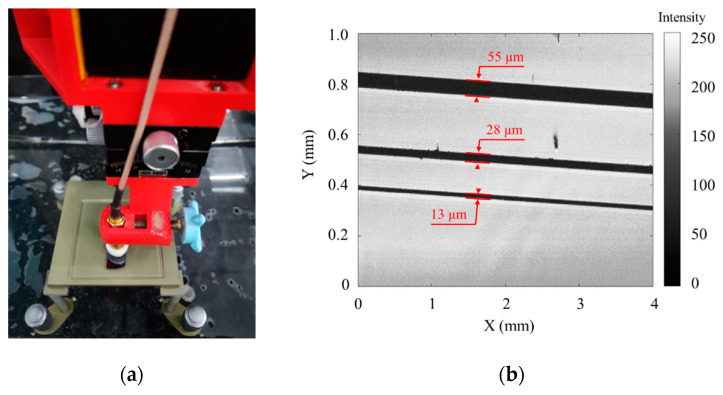
(**a**) Silicon wafer imaging testing. (**b**) The ultrasonic image of slots on silicon wafer.

Silicon wafers with three slots were imaged by SAM (Figure 8a). The widths of three slots were 55 μm, 28 μm, and 13 μm respectively. As shown in Figure 8b, the three slots were distinguished clearly. The high lateral resolution was demonstrated.

## 4. Discussion

In this paper, silicon wafer was chosen as the material of UHF acoustic lens because of its high acoustic velocity, low acoustic attenuation and excellent machinability. Silicon lenses with an aperture of 892 μm and a depth of 252 μm were prepared by ultraprecision machining with a maximum error of 8 μm. Silicon lenses were used to fabricate UHF ultrasonic transducers with a center frequency of 157 MHz and a −6-dB bandwidth of 52%. The focal distance of the transducers was 736 μm, and the F-number was about 0.82. The transducers had a lateral resolution of 11 μm and could distinguish the 13 μm slots on the silicon wafer clearly. In addition, a short pulse duration of 8 ns was achieved, which represents high axial resolution.

In previous research, etching has been widely used in the fabrication of acoustic lenses based on silicon wafers. Acoustic lenses usually should have a smooth surface and larger geometry sizes than semiconductor devices. Etching is divided into wet etching and dry etching. Wet etching [18] has a faster etching rate, but when materials have poor anisotropy, smooth surfaces are difficult to prepare by wet etching. Though dry etching [14] can produce smooth surfaces, large size etching is time-consuming and expensive using dry etching. Moreover, for dry etching, it is preferable to use vertical etching to spherical etching. Thus, although etching is a common method for processing silicon wafer in the semiconductor field, it is not the best choice for preparing acoustic lenses. Ultra-precision machining relies on a micron-sized ball nose milling cutter, which can be used to machine any spherical morphology in silicon lenses. As shown in Table 4, ultra-precision machining has the advantage of low cost and shorter processing time. As etching involves a series of steps including coating, exposure, development, etching, striping, it is more complex than ultraprecision machining. Furthermore, ultraprecision machining is independent of the anisotropy of silicon wafer which decreases the difficulty of processing. Therefore, ultraprecision machining should have a bright future in the preparation of UHF acoustic lenses.

For UHF applications of biomedical imaging, the frequency limit is a key problem. The preparation of silicon lenses for use in higher frequency imaging will continue to be researched. Sapphire [27] is a more suitable material for preparing UHF acoustic lenses because of its higher acoustic velocity and lower acoustic energy attenuation than silicon, but it is more difficult to process. The preparation of sapphire lenses will be another challenging target.

## Figures and Tables

**Table 1:** The parameters of the silicon wafer.

Size (feet)	Thickness (μm)	Type	Orientation	Resistivity (Ω·cm)	Surface Treatment
2	500	N	100	1–10	single-side polishing, no oxide film

**Table 2:** The parameters of materials in the COMSOL simulation.

	Velocity (m/s)	Density (kg/m^3^)	Acoustic Impedance (MRayl)
LiNbO_3_	7360	4688	34.5
Si	8430	2340	19.8
E-solder 3022	1850	3200	5.9
Water	1540	1000	1.5

**Table 3:** The performance parameters of the UHF ultrasonic transducer.

	Center Frequecy (MHz)	−6-dB Bandwidth (%)	Pulse Duration (ns)	Focal Distance (μm)
Silicon lens Transducer	157	52	8	736

**Table 4:** The advantages of ultraprecision machining.

Method	Cost	Time	Complexity	Difficulty
Ultraprecision machining	Low	Short	Single step	Easy
Etching	High	Long	A series of steps	Hard

## Data Availability

Not applicable.
